# N-acetyl cysteine restores the fertility of vitrified–warmed mouse oocytes derived through ultrasuperovulation

**DOI:** 10.1371/journal.pone.0224087

**Published:** 2019-10-22

**Authors:** Ayumi Mukunoki, Toru Takeo, Naomi Nakagata

**Affiliations:** Division of Reproductive Engineering, Center for Animal Resources and Development, Kumamoto University, Honjo, Chuo-ku, Kumamoto, Japan; Universita degli Studi dell'Insubria, ITALY

## Abstract

Oocyte cryopreservation is useful for preserving fertility and storing genetic resources. However, the small number of oocytes acquired using conventional treatment to induce superovulation and the reduction of fertility due to cryopreservation represent significant problems. Herein, we vitrified the oocytes derived through high-yield superovulation using inhibin antiserum and equine chorionic gonadotropin (IAS + eCG: IASe) and examined the yield of cryopreserved oocytes and survival rates relative to those of vitrified–warmed mouse oocytes derived through conventional superovulation using equine chorionic gonadotropin (eCG). Furthermore, we investigated the effects of N-acetyl cysteine on the fertility and developmental potential of vitrified–warmed oocytes derived using IASe. Compared with eCG, IASe increased the yield of cryopreserved oocytes and achieved equivalent survival rates. N-acetyl cysteine (0.5 mM) increased the fertilization rate of vitrified–warmed oocytes derived using IASe. Vitrification decreased thiol levels in the zona pellucida (ZP), while warming followed by N-acetyl cysteine treatment increased free thiol levels in ZP. Moreover, N-acetyl cysteine treatment recovered zona hardening by cleaving disulfide bonds and promoting the expansion of ZP. Two-cell embryos derived via *in vitro* fertilization using N-acetyl cysteine developed into normal pups through embryo transfer. Therefore, we developed an efficient technique for the production of cryopreserved oocytes using IASe through superovulation and found that N-acetyl cysteine improves the fertility of vitrified–warmed oocytes by cleaving the disulfide bonds and promoting the expansion of ZP.

## Introduction

Oocyte cryopreservation is widely used for preserving genetic resources to breed animals for medical research and to support the infertility treatments in human [1]. In cryopreservation, oocytes are collected from donors treated with hormones or drugs to induce superovulation and preserved with cryoprotective agents under liquid nitrogen. After warming the oocytes, embryos are produced through intracytoplasmic sperm injection (ICSI) or *in vitro* fertilization (IVF), and the produced embryos are subsequently preserved or developed.

Mouse oocytes can be cryopreserved using vitrification and slow freezing methods [2–6]. We previously developed a simple vitrification method using a highly concentrated vitrification solution [2, 7–9], which achieves a relatively high survival rate of mouse oocytes after cryopreservation. However, the number of embryos and pups derived from vitrified oocytes is insufficient because of the low yield of oocytes induced by conventional superovulation techniques (20–30 oocytes/mouse) and the reduced fertility of vitrified–warmed oocytes used for conventional IVF.

To overcome this limitation, we developed a technique involving the co-administration of inhibin antiserum and equine chorionic gonadotropin (IAS + eCG: IASe), termed ultrasuperovulation, which produces approximately 100 oocytes from a single 4-week-old mouse [10]. Furthermore, we found that cysteine analogs such as N-acetyl cysteine (NAC), L-cysteine, D-cysteine, and reduced glutathione enhance the success of IVF employing freshly harvested oocytes as well as frozen-thawed or cold-stored mouse sperm [11–13]. Ultrasuperovulation combined with IASe and IVF using NAC may overcome the shortcomings of oocyte cryopreservation. To the best of our knowledge, the efficacies of techniques employing ultrasuperovulation and improved IVF using cryopreserved oocytes have not been evaluated.

In this study, we compared the yields and viabilities of vitrified–warmed oocytes prepared using eCG or IASe and evaluated the effects of NAC on the fertilization rate of vitrified–warmed oocytes. Moreover, we examined thiol levels in the zona pellucida (ZP) by evaluating the fluorescence intensity of a thiol-selective compound and expansion of ZP by dimensional area treated with or without NAC after vitrifying and warming. Finally, we evaluated the development of NAC-treated vitrified–warmed oocytes using embryo culture and transfer.

## Materials and methods

### Mice

Male and female C57BL/6J mice (CLEA Japan), 12- and 4-week-old, respectively, were used as sperm and oocytes donors. Female ICR mice (CLEA Japan) aged 12 weeks were used as the recipients of two-cell embryo transfer. The mice were housed in a specific-pathogen-free room with a 12/12-h dark/light cycle (illumination from 7:00 to 19:00) at 22°C ± 1°C and provided food and water *ad libitum*. The Animal Care and Use Committee of the Kumamoto University School of Medicine approved the protocols of all experiments using animals.

### Media

IAS was derived from goats immunized with an inhibin-specific synthetic peptide coupled to keyhole limpet hemocyanin [14]. Oocyte vitrification was performed using 1 M dimethyl sulfoxide (DMSO) and a solution of 2 M DMSO, 1 M acetamide, and 3 M propylene glycol (DAP213) [8]. A solution of 0.25 M sucrose (37°C) was used to warm the vitrified oocytes. High-calcium human tubal fluid (mHTF) containing NAC was used for IVF [15, 16]. Sperm were preincubated in Toyoda–Yokoyama–Hosi medium supplemented with methyl-beta-cyclodextrin (cTYH) [17, 18]. Embryos were cultured in potassium simplex medium with amino acids (KSOM/AA) [19]. All media were stored at 4°C. Fertilization and sperm and embryo culture media were preincubated in an incubator for 30 min at 37°C in an atmosphere containing 5% CO_2_.

### Superovulation and oocyte collection

Superovulation was performed as previously described [10]. Female mice were intraperitoneally injected with 7.5 IU eCG (ASKA Pharmaceutical Co. Ltd, Japan) or IASe (3.75 IU eCG and 0.1 mL IAS). After 48 h, the mice were injected with 7.5 IU hCG (ASKA Pharmaceutical Co. Ltd, Japan). Next, 17 h after hCG administration, the mice were euthanized by cervical dislocation, and oviducts were immediately collected and transferred to liquid paraffin covered by a drop of fertilization medium. A dissection needle was used to collect oocytes from the ampullae, which were placed on a 200-μL drop of mHTF. IASe or eCG ovulated 96% or 83% of morphologically normal oocytes.

### Oocyte vitrification

Oocyte vitrification was performed as previously described [2]. Briefly, 0.1% hyaluronidase in mHTF was used to remove the cumulus cell. Cumulus-free oocytes were transferred into mHTF containing 20% fetal bovine serum and cultured for 10 min in a CO_2_ incubator. The oocytes were transferred onto a 100-μL drop of 1 M DMSO and divided into groups of 20–30, transferred into a cryotube containing 5- μL DMSO solution, and placed on ice. After 5 min, 45-μL of DAP213 was added to the cryotube containing oocytes, which was placed on ice for 5 min and then in liquid nitrogen. Vitrified oocytes were warmed in 0.25 M sucrose at 37°C. Specifically, liquid nitrogen was discarded from cryotube, and the cryotubes were allowed to stand at room temperature for 30 s. Then, 0.25 M sucrose was added to cryotubes, and vitrified oocytes were warmed quickly via pipetting. The oocytes were washed three times with 100-μL drops of mHTF, and the morphologically normal oocytes were counted.

### IVF

Vitrified–warmed oocytes were incubated in a 200-μL drop of mHTF containing NAC for 1 h at 37°C in a CO_2_ incubator. Male mice were euthanized by cervical dislocation. Sperm were collected from the cauda epididymides and incubated in a 100-μL drop of cTYH for 1 h at 37°C in a CO_2_ incubator. The sperm suspension (3 μL) was added to the fertilization medium containing the vitrified–warmed oocytes. The final concentration of sperm in the fertilization medium was 800–2,000 sperm/μL. After 3 h, the oocytes were inseminated and washed with 80-μL drops of mHTF. Two-cell embryos were counted after 24 h, and the percent fertilization rates were calculated as the total number of two-cell embryos divided by the total number of unfertilized oocytes.

### Thiol levels in ZP

Thiol levels in the ZP were measured as previously described [13]. Alexa Fluor 488 C5 maleimide (AFM, Life Technologies) is a thiol-selective fluorescent compound that can detect free thiols in ZP. Thiol levels in ZP were analyzed based on fluorescence intensity of AFM using image analysis software (BZ-H2A version 1.42, Keyence Co.). AFM was dissolved in phosphate-buffered saline with polyvinyl alcohol (PBS–PVA) and stored at −20°C. Fresh or vitrified–warmed oocytes were incubated with or without NAC, washed two times with 100-μL drops of PBS–PVA, and incubated with AFM (0.1 mg/mL) at room temperature in the dark. After 30 min, oocytes were washed three times with 100-μL drops of PBS–PVA and observed using a fluorescence microscope (Biorevo BZ-9000, Keyence Co.). The fluorescence intensity of ZP was measured using image analysis software (BZ-H2A version 1.42, Keyence Co.). The relative fluorescence intensity was calculated as the value of vitrified–warmed oocytes preincubated with or without NAC divided by the value of fresh oocytes.

### ZP expansion

ZP expansion was examined by measuring the cross-sectional area of oocytes using image analysis software (BZ-H2A version 1.42, Keyence Co.). The percent relative cross-sectional area was calculated as the value of vitrified–warmed oocytes cultured with or without NAC divided by the value of oocytes cultured without NAC.

### Embryo culture and transfer

The development of two-cell embryos derived from vitrified–warmed oocytes was evaluated using *in vitro* culture and embryo transfer. The two-cell embryos were washed three times with KSOM/AA and cultured in a CO_2_ incubator blastocyst formation. Two-cell embryos (n = 10) were then transferred into each oviduct of a pseudopregnant mouse using a vaginal plug [20]. The number of pups was counted after 19 days.

### Statistical analysis

Statistical analysis was performed using Prism version 7.02 (GraphPad Software). The results are expressed as mean and standard deviation. Comparisons were performed using the analysis of variance following arcsine transformation of the percentages. P < 0.05 was considered statistically significant.

## Results

### IASe produced high yield of surviving oocytes after vitrifying and warming

**Fig 1:**
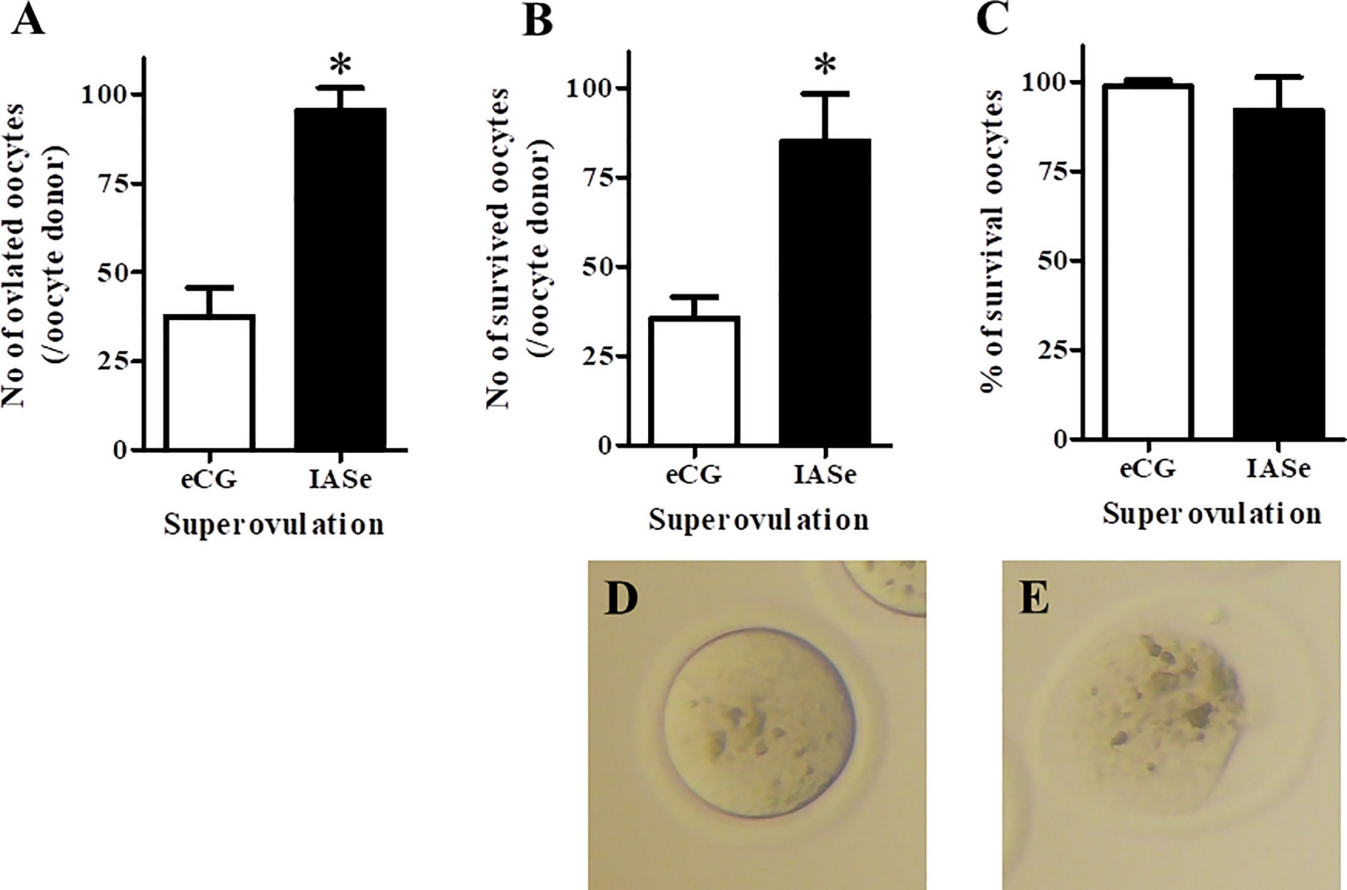
Effect of superovulation induced by eCG or IASe treatment on the viability and yield of vitrified–warmed oocytes. A) The number of ovulated oocytes per oocyte donor was counted after removing the cumulus cells. B) The number of surviving oocytes per oocyte donor was counted after vitrifying and warming. C) The rate of survival of oocytes (%) was calculated as the number of morphologically normal oocytes divided by the sum of morphologically normal and dead oocytes × 100. Morphologically normal oocyte (D) and dead oocyte (E) after vitrifying and warming are shown. The results are expressed as mean ± SD (n = 5). *P < 0.05 compared with eCG.

To examine the productivity of surviving oocytes under IASe treatment after vitrifying and warming, we compared the effects of eCG and IASe on the number of ovulated oocytes, the number of surviving oocytes after vitrifying and warming, and the rate of survival. The number of IASe-treated ovulated oocytes was approximately three times higher than that of eCG-treated ovulated oocytes (Fig 1A, eCG: 35.8 oocytes/female *vs*. IASe: 93.6 oocytes/female). After vitrifying and warming, IASe treatment showed a higher number of surviving oocytes per oocyte donor than eCG treatment (Fig 1B, eCG: 35.4 oocytes/female *vs*. IASe: 85.0 oocytes/female). The survival rate IASe-treated vitrified–warmed oocytes was equal to that of eCG-treated vitrified–warmed oocytes (Fig 1C, eCG: 98.9% *vs*. IASe: 92.4%). IASe was effective in terms the yield of cryopreserved oocytes without any adverse effects on vitrification. Therefore, all subsequent experiments were performed using IASe-treated oocytes.

### NAC treatment enhanced the fertility of vitrified–warmed oocytes derived using IASe

**Fig 2:**
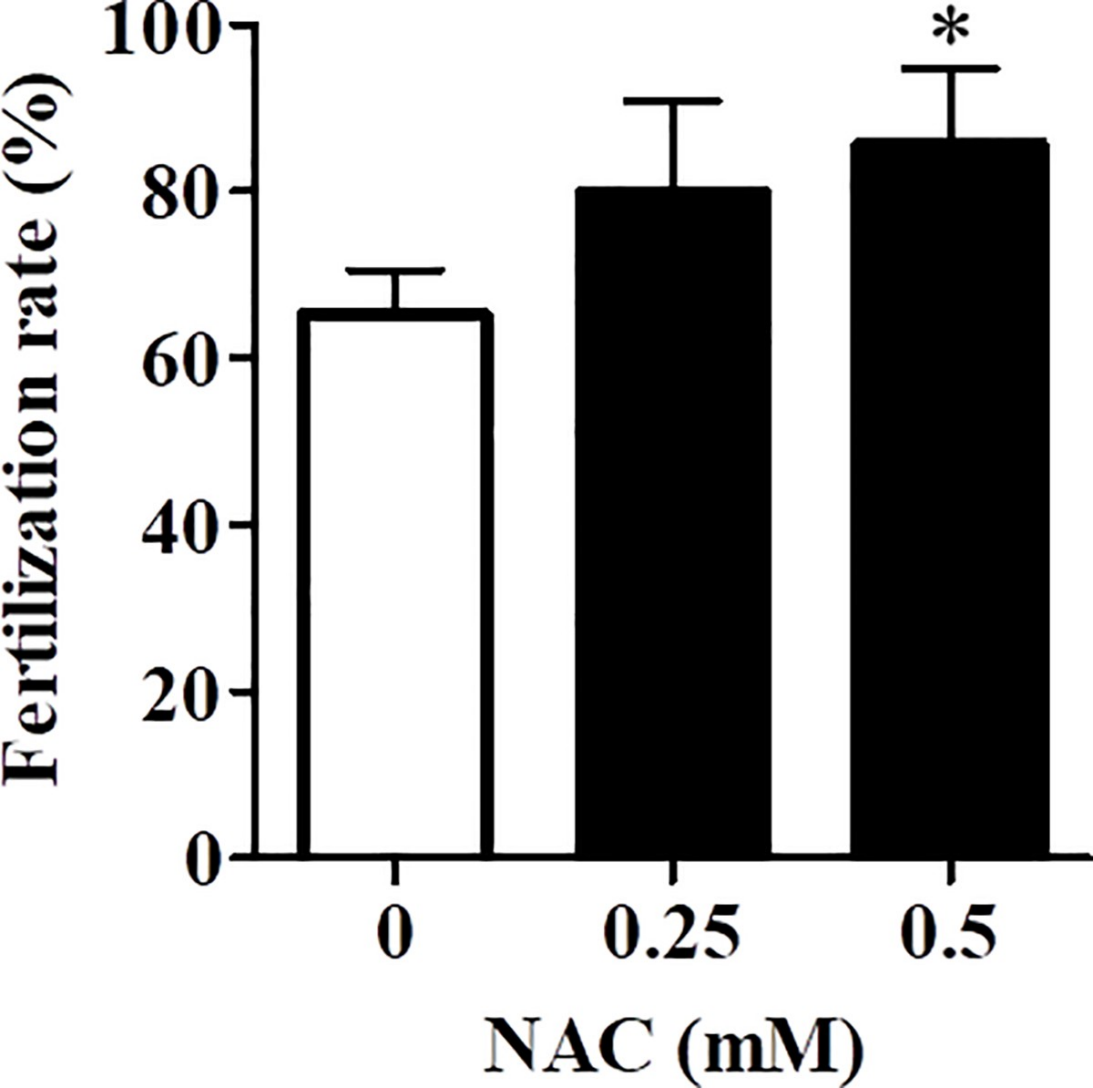
Effect of NAC on the fertilization rate of vitrified–warmed oocytes. The fertilization rate was calculated as follows: total number of two-cell embryos divided / (the total number two-cell embryos + unfertilized oocytes) × 100. Results are expressed as mean ± SD (n = 6). *P < 0.05 compared with control (0 mM NAC).

To evaluate the effects of NAC on the fertility of vitrified–warmed oocytes derived using IASe, oocytes were incubated with mHTF containing NAC (0.25 mM or 0.5 mM) for 1 h and then incubated them with fresh sperm. The fertilization rate of vitrified–warmed oocytes increased with increased concentration of NAC (Fig 2, 0 mM: 65.5% *vs*. 0.25 mM: 80.5% and 0.5 mM: 85.9%). In particular, 0.5 mM NAC-treated oocytes showed a higher fertilization rate than control oocytes (0 mM NAC).

### Thiol levels in ZP decreased following cryopreservation

**Fig 3:**
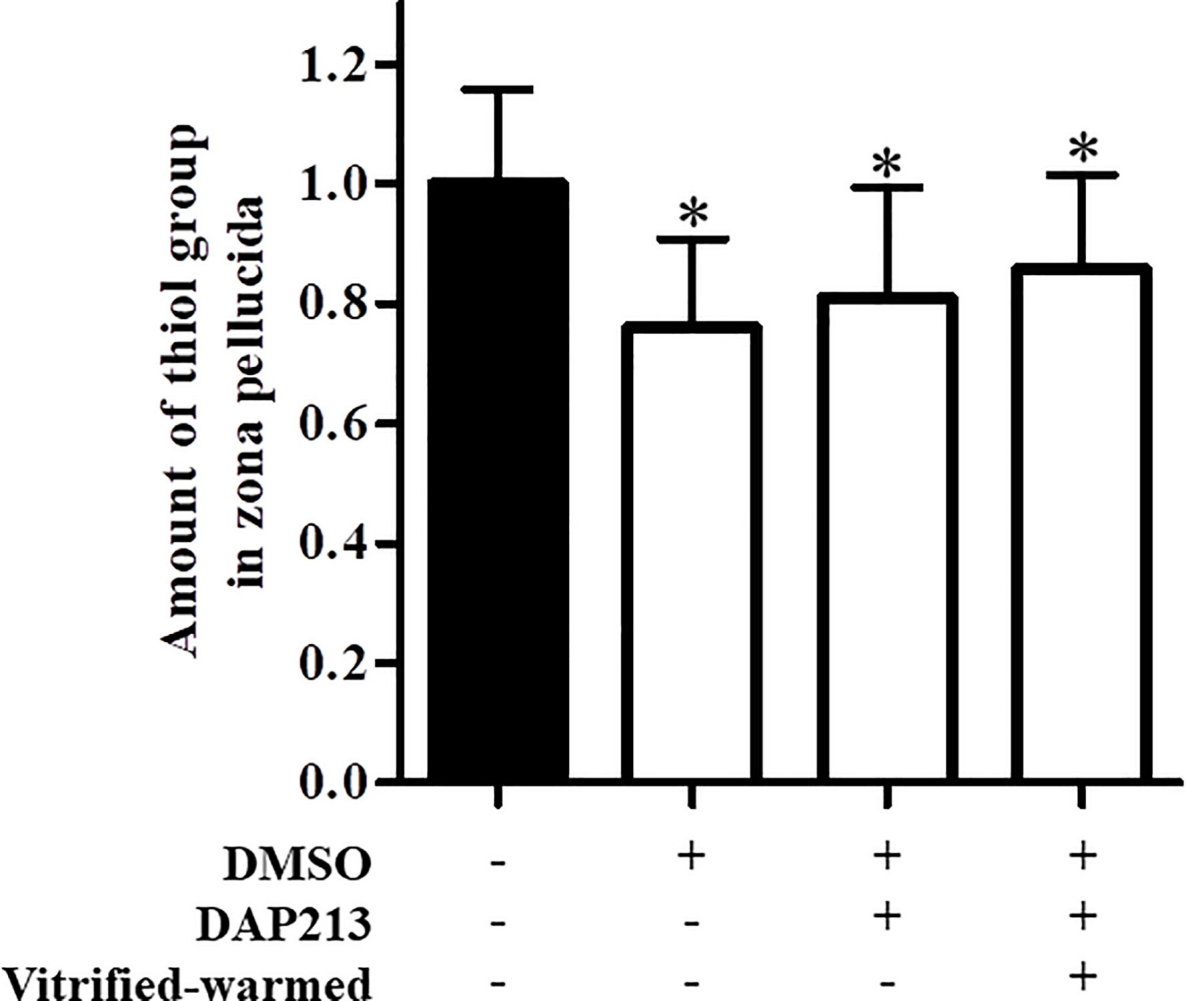
Thiol levels following treatment with cryoprotectants. Relative fluorescence intensity was calculated as follows: fluorescence intensity of vitrified–warmed oocytes precultured with NAC / fluorescence intensity of fresh oocytes. Results are expressed as mean ± SD (fresh: n = 85, DMSO: n = 85, DAP213: n = 52, thawed: n = 86). *P < 0.05 compared with fresh oocytes [DMSO (−), DAP213 (−), vitrified–warmed (−)].

To explore the mechanism underlying decreased fertilization rate of vitrified–warmed oocytes, we examined thiol levels in ZP following vitrification. Oocyte cryopreservation reportedly induces zona hardening via disulfide bond formation. Thiol levels of oocytes treated with 1 M DMSO, DAP213, and thawing were significantly decreased compared with those fresh oocytes (Fig 3). Therefore, DMSO and DAP213 treatments as well as vitrifying and warming induced zona hardening via disulfide bonds formation.

### NAC treatment elevated thiol levels and induced ZP expansion

**Fig 4:**
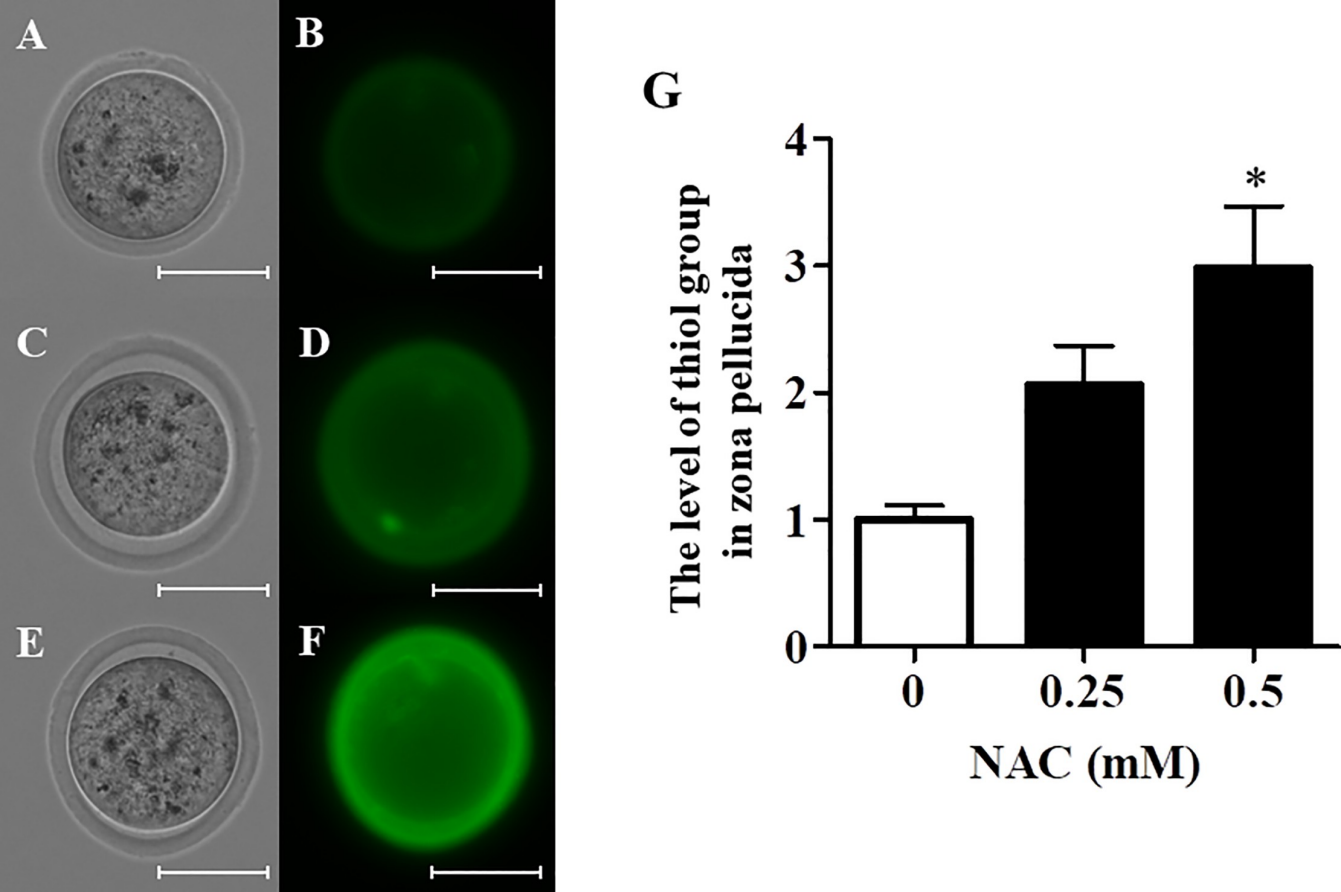
Effect of NAC on thiol levels in vitrified–warmed oocytes. The level of thiol group of vitrified warmed oocytes treated with or without NAC was measured by fluorescence intensity of the thiol-selective fluorescent compound AFM (31–34 oocytes/experiment). The oocytes were shown (A: 0 mM, C: 0.25 mM, E: 0.5 mM: bright field, B: 0 mM, D: 0.25 mM, F: 0.5 mM: fluorescence, scale bar shows 50 μm). G) Thiol levels in the ZP were calculated as follows: Fluorescence intensity of each group / Fluorescence intensity of 0 mM NAC) × 100. Results are expressed as mean ± SD (n = 4). *P < 0.05 compared with control (0 mM NAC).

**Fig 5:**
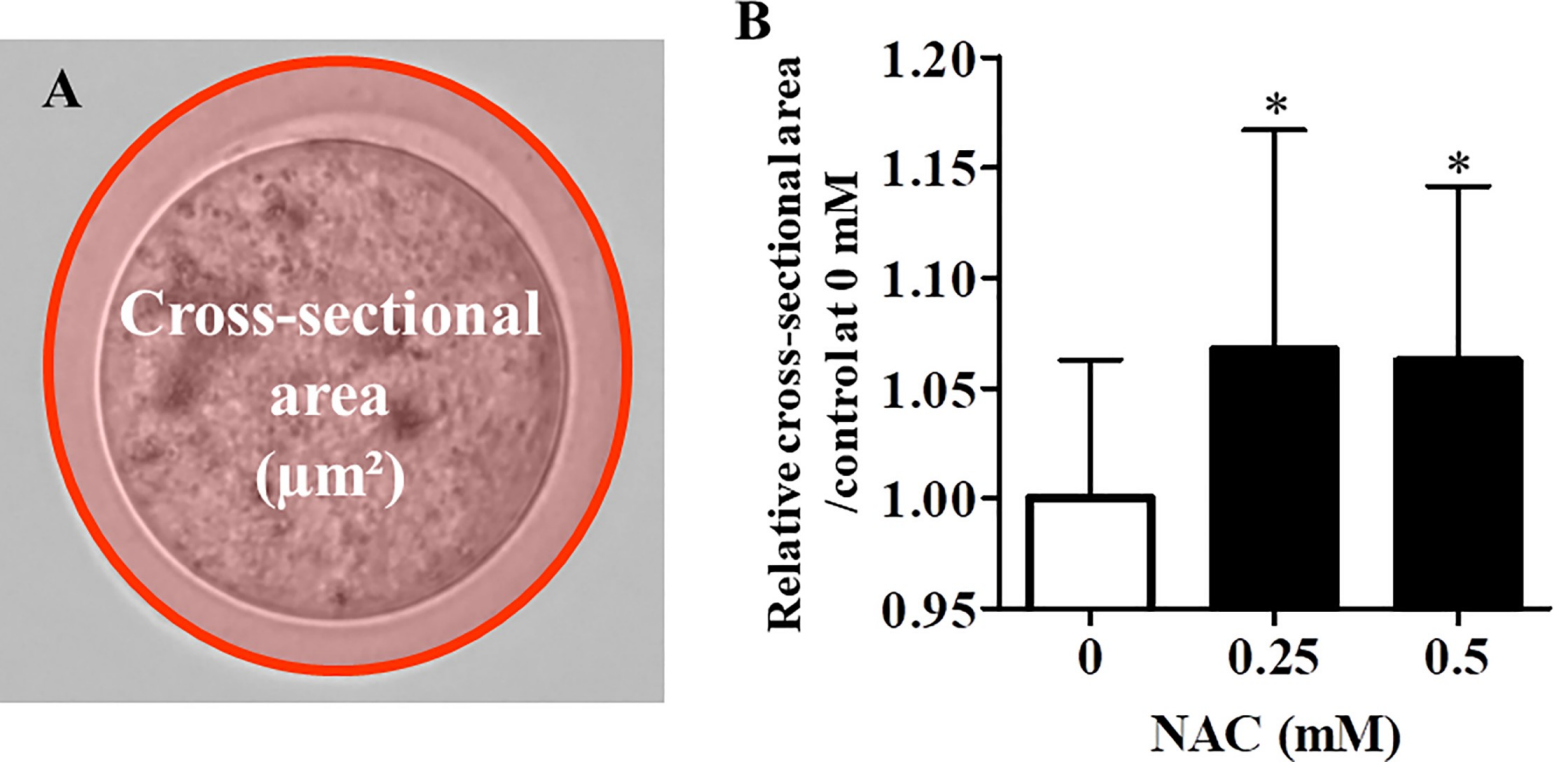
Effect of NAC on zona pellucida expansion in vitrified–warmed oocytes. A) Cross-sectional area of the zona pellucida was measured (31–34 oocytes/experiment). B) The extent of expansion of the zona pellucida was calculated as follows: (cross-sectional area of each group / cross-sectional area in 0 mM NAC) × 100. Results are expressed as mean ± SD (n = 4). *P < 0.05 compared with control (0 mM NAC).

To examine variations of thiol levels in ZP, vitrified–warmed oocytes were incubated in mHTF containing NAC for 1 h and then with the thiol-selective fluorescent compound AFM. NAC treatment increased thiol levels in ZP (Fig 4A–4G). These data suggest NAC cleaved disulfide bonds in ZP after vitrifying and warming. In addition, NAC treatment induced ZP expansion in vitrified-warmed oocytes (Fig 5A and 5B).

### Vitrified–warmed oocytes derived using IASe developed into normal pups

The developmental ability of vitrified–warmed oocytes derived using IASe was assessed *in vitro* and after embryo transfer. The embryos derived from vitrified–warmed oocytes developed into blastocysts and normal pups (Tables 1 and 2).

**Table 1:** Effects of NAC on the development of vitrified–warmed oocytes.

NAC (mM)	Number of inseminated oocytes	Number of two-cell embryos (%)	Number of four-cell embryos (%)	Number of morulae (%)	Number of blastocysts (%)
0	263	165 (65.5±5.5)	156 (94.5 ± 3.9)	150 (90.9 ± 9.1)	138 (83.6 ± 8.8)
0.25	261	207 (80.5±11.0)	199 (96.1 ± 5.0)	193 (93.2 ± 5.6)	169 (81.6 ± 7.5)
0.50	264	219 (85.9±9.1*)	214 (97.7 ± 2.8)	209 (95.4 ± 3.1)	181 (82.6 ± 13.5)

Vitrified–warmed oocytes were inseminated and fertilization rate was calculate as follows: Number of two-cell embryos / Number of inseminated oocytes) × 100. After IVF using vitrified–warmed oocytes, two-cell embryos were cultured in KSOM/AA. Developmental rate was calculated as follows: (Number of four-cell embryos, morulae, or blastocysts / Number of two-cell embryos) × 100. Results are expressed as mean ± SD (n = 6).

*P < 0.05 compared with control (0 mM).

**Table 2:** The birth rate of vitrified–warmed oocytes derived from IVF with NAC.

NAC (mM)	Oocyte	Number of transferred two-cell embryos	Number of recipients	Number of live pups (%)
0	Fresh	80	4	31 (38.8 ± 16.5)
0	Vitrified–warmed	80	4	32 (40.0 ± 15.8)
0.5	Vitrified–warmed	80	4	37 (46.3 ± 11.1)

Two-cell embryos were transferred in each oviduct of the recipient mice (20 embryos/recipient), and the number of pups was recorded after 19 days. Two-cell embryos derived from fresh oocytes were used as controls. The birth rate was calculated as follows: (Number of the pups / Number of transferred two-cell embryos) × 100. Results are expressed as mean ± SD (n = 4).

## Discussion

In this study, we demonstrated that ultrasuperovulation employing IASe significantly increased the yields of ovulated and cryopreserved oocytes. The survival rates of IASe- and eCG-derived oocytes were comparable, and the reduced fertilization rate was recovered by NAC treatment. Moreover, NAC treatment rescued depleted thiol levels in ZP following exposure to cryoprotectants. Embryos derived using ultrasuperovulation employing IASe and IVF with NAC produced normal pups. Therefore, our findings provide convincing evidence that ultrasuperovulation employing IASe and IVF with NAC enhances the productivity of cryopreserved oocytes and embryos derived from vitrified–warmed oocytes.

Oocyte cryopreservation is useful for preserving genetic resources and for breeding animals using ICSI or IVF and embryo transfer. Researchers can design genetically engineered mouse models tailored to their objectives. Moreover, oocyte cryopreservation can reduce the cost and space required for breeding. In addition, techniques used for the oocyte cryopreservation of mammalian species are continuously improving [21]. For instance, the viability and fertility of cryopreserved oocytes are affected by cryoprotectants such as glycerol, propylene glycol, and DMSO as well as by the cooling and warming rates, devices employed, and storage temperature [22]. Optimization of the use of these reagents is effective to improve the survival and fertilization rates of cryopreserved oocytes. Based on these data, the use of oocytes cryopreservation is popular among researchers who use the genetically engineered mice worldwide. However, there remain some shortcomings in terms of the limited yield of cryopreserved oocytes and their low fertilization rates.

To overcome these limitations, we combined the two techniques of ultrasuperovulation using IASe and IVF with NAC. The combined technique produced 3-times higher yield and >80% higher IVF fertilization rate of cryopreserved oocytes than the conventional techniques. These results suggest that such optimized techniques can enhance the productivity and availability of cryopreserved oocytes. The superovulation technique is important to maximize the oocyte yield for producing cryopreserved stocks. The former reduces the number or cycles of oocyte collection and enables rapid storage of oocytes.

The fertility of IASe-derived oocytes and their ability to mature are the same as those of normal oocyte [10]. Moreover, IASe treatment could increase the yield of ovulated oocytes of major inbred (A/J, BALB/cByJ, C3HeJ, DBA/2J, and FVB/NJ) and outbred (CD1) mouse strains [23]. In our experiments, IASe-derived oocytes tolerated cryopreservation and recovered their fertility following IVF with NAC. The combined technique of ultrasuperovulation using IASe, oocyte cryopreservation, and IVF using NAC may improve the archiving of germ cells derived from diverse strains of female mice.

Cryopreservation reduces the fertility of oocytes, primarily due to cryo-induced zona hardening [24–26]. During cryopreservation, transient calcium influx in oocytes triggers the exocytosis of cortical granule and hardens the zona [27]. During zona hardening, free thiols in ZP interact to form intra- and inter-molecular disulfide bonds among proteins, thereby altering the ZP structure [28]. DMSO, a cryoprotectant used for oocytes cryopreservation, induces cortical granule exocytosis at 37°C, which is alleviated at 4°C [29, 30]. In this study, we demonstrated that DMSO facilitated the formation of disulfide bonds in ZP (Fig 3). Our findings indicate that DMSO may induce cortical granule exocytosis and ZP oxidation as well as promote cysteine cross-linkage.

We previously found that GSH and the thiol analogs L-cysteine, D-cysteine, and NAC cleave disulfide bonds in ZP, thus increasing the fertilization rates achieved using IVF with fresh oocytes and frozen-thawed or cold-stored sperm [11, 13]. In the present study, NAC similarly restored the fertility of vitrified–warmed oocytes (Fig 2) and increased the levels of free thiols in ZP (Fig 4C). These findings indicate that NAC can recover cryo-induced zona hardening via disulfide bonds cleaving, which in turn can improve the fertility of vitrified–warmed oocytes.

NAC enhances the cryopreservation of mouse oocytes. For instance, after vitrification, 1.0 mM NAC improved mitochondrial activity and vitrified–warmed oocyte maturation [31]. When mouse oocytes were cryopreserved at the germinal vesicle stage, 1.5 mM NAC added to the media for vitrification and *in vitro* maturation enhanced maturation by inhibiting reactive oxygen species generation [32]. Similarly, the antioxidant resveratrol recovered the ability of vitrified–warmed mouse oocytes to mature by reducing oxidative stress, which alleviates abnormalities of mitochondrial distribution [33]. However, thiol levels in ZP and zona expansion of the oocytes vitrified with cryoprotectants containing NAC were not confirmed, and the additional research is warranted.

In this study, two-cell embryos produced by IVF using IASe-derived oocytes after vitrifying and warming developed into blastocysts and pups (Tables 1 and 2). IASe and NAC treatment did not show detectable adverse effects on the development of cryopreserved oocytes. However, there is at least one report, to our knowledge, that the superovulation method, oocyte vitrification, and *in vitro* embryo culture induce epigenetic modifications of early embryos [34]. Further investigations of epigenetic modifications are imperative to evaluate the safety of these techniques for agricultural and medical use.

Reproductive technology has become increasingly important in the fields of biotechnology, agriculture, medicine, and environment conservation. For example, mice serve as models for advanced applications of reproductive technology. At least 54,600 mouse strains have been archived as cryopreserved embryos and sperm [35, 36], which are efficiently distributed to research institutes worldwide. Mice can be rapidly produced and analyzed and then re-archived [37, 38]. Oocyte cryopreservation facilitates the use of archived female germ cells in diverse applications such as introducing gene modifications using IVF with genetically modified sperm or for genome editing after IVF. Mice can serve as suitable models to demonstrate the applications of reproductive technology.

In conclusion, we developed a system for the efficient production of embryos and mice from vitrified–warmed oocytes using ultrasuperovulation and IVF with NAC. Vitrification of oocytes reduces the space required for breeding mice as well as the cost of feeding mice, thus enabling IVF to be performed immediately after warming vitrified oocytes. Furthermore, ultra-superovulation can greatly increase the number of vitrified oocytes, which can reduce the number of mice required for experiments (i.e., the 3Rs “Reduction of the number of experimental animals”). We believe these techniques will improve animal experimentation and promote research focused on developing new therapeutics and medicines.
